# Subsequence and distant supervision based active learning for relation extraction of Chinese medical texts

**DOI:** 10.1186/s12911-023-02127-1

**Published:** 2023-02-14

**Authors:** Qi Ye, Tingting Cai, Xiang Ji, Tong Ruan, Hong Zheng

**Affiliations:** School of Information Science and Technology, East China University of Science and Technology, Shanghai, 200237 China

**Keywords:** Active learning, Sequence tagging, Relation extraction, Distant supervision, Medical texts

## Abstract

In recent years, relation extraction on unstructured texts has become an important task in medical research. However, relation extraction requires a large amount of labeled corpus, manually annotating sequences is time consuming and expensive. Therefore, efficient and economical methods for annotating sequences are required to ensure the performance of relational extraction. This paper proposes a method of subsequence and distant supervision based active learning. The method is annotated by selecting information-rich subsequences as a sampling unit instead of the full sentences in traditional active learning. Additionally, the method saves the labeled subsequence texts and their corresponding labels in a dictionary which is continuously updated and maintained, and pre-labels the unlabeled set through text matching based on the idea of distant supervision. Finally, the method combines a Chinese-RoBERTa-CRF model for relation extraction in Chinese medical texts. Experimental results test on the CMeIE dataset achieves the best performance compared to existing methods. And the best F1 value obtained between different sampling strategies is 55.96%.

## Introduction

With the development of society, national health awareness is constantly improving, and the construction of intelligence and information in the medical field is also steadily advancing. At present, a large amount of medical texts have been accumulated in the existing medical information system, including medical literature, Electronic Health Records (EHRs), and so on [1]. A lot of important information are stored in an unstructured form, which is difficult for computers to understand and process. Through Natural Language Processing (NLP) technology, medical text can be analyzed and transformed into high-quality knowledge that is convenient for computer processing, thus providing valuable data resources for medical workers and researchers. Relation extraction(RE) refers to the extraction of relational triplet between entity pairs from medical text [2, 3]. The triplet is represented as “(head entity, relationship, tail entity)” [4]. Relation extraction plays an important role in NLP and knowledge graphs (KG) [5].

According to the model structure, the relation extraction model can be divided into two types: pipeline and joint extraction. The pipeline model treats entity recognition and relationship classification as two independent subtasks. This will cause the problem of redundant entity inference and propagation errors [6]. The joint extraction model regards relation extraction as a whole task, and completes entity recognition and relationship classification at the same time [7]. The joint extraction structure can be divided into parameter sharing type and sequence tagging type according to their implementation methods. The parameter sharing type models the two subtasks separately, which avoids propagating errors through sharing model parameters between two subtasks. However, building two models at the same time will waste a lot of computing resources. Recently, sequence tagging for joint entity and relation extraction has been proposed which can void the propagation of errors and save the computing resources, and thus it has recently become a major focus on relation extraction research [8, 9].

The sequence tagging method transforms entity-relation extraction into a sequence tagging task by designing a labeling system that contains both entity and relation information. It typically employs neural network models to learn the contextual representation of text and then model the labeled sequences through decoders [10]. The effectiveness of models usually relies on large scale manually annotated corpus sets. However, tagging medical texts is more challenging than other tasks such as text segmentation and named entity recognition(NER). On the one hand, most of the medical relation extraction datasets are derived from medical literature, which is highly professional and theoretical, making it difficult for annotators without substantial medical knowledge to understand the obscure expertise in the literature. On the other hand, relationship extraction is a high-level task in the NLP field, and the dataset for this task requires annotation of both entities and relationships between entities, which further increases the workload of the medical relationship extraction annotation task.

To reduce the human annotation work in sequence tagging, active learning methods are applied to this task. Active learning starts with a small number of seed labeled sequences and iteratively trains the model on the dataset that has already been annotated. It then iteratively selects sequences with high annotation values for expert annotation, and It can help to some extent with the issue of time-consuming and expensive manual annotation of medical texts.

However, traditional active learning [7, 11, 12] uses a whole sentence as the sampling unit, which has some drawbacks for the task of medical relationship extraction. From the perspective of the sample, different subsequences in the sample have different annotation values, i.e. there is heterogeneity within each instance. The model may be able to make correct judgments on the labels of subsequences that have been labeled by the expert, while not so sure about those have not been labeled. Therefore, sampling at the full sentence level may lead to a waste of annotation resources. Moreover, from the annotator’s point of view, annotating long sentences may lead to physical and mental fatigue, which will affect the quality and efficiency of annotation.

In this work, we try to alleviate the problem of extensive manual annotation and cost in Chinese medical texts relation extraction tasks. The introduction of subsequence annotation not only improves the efficiency of annotation, but also improves the performance of the relational extraction model. This essay builds Chinese-RoBERTa-CRF as a model for extracting the relationships between entities. The model is Chinese BERT with Whole Word Masking, which is more suitable for chinese text processing. The model fully captures the contextual representation of the text through a two-layer Transformer structure. And CRF decoder is used to model the dependencies between labels, thus it enables accurate identification of relationships between entities in the Chinese medical texts. Experiments are presented on the CMeIE dataset to evaluate the effectiveness of our method. It achieves the highest F1 value compared to existing benchmark methods. Contributions of this paper are:

- We propose a subsequence and distant supervision based active learning (SDSAL) method for Chinese medical texts relation extraction and experiment shows it could be beneficial for annotation efficiency by selecting information-rich subsequences in the sentence.
- We investigate dictionary matching to pre-label the unlabeled set based on distant supervision, propagating their labels to other texts, which can further improve the labeling efficiency and reduce annotation costs.
- We suggest using a Chinese-RoBERTa-CRF model with active learning, which has been demonstrated to be particularly effective for relation extraction tasks.

## Related work

**Table 1:** Comparison between different RE methods

Method type	Characteristics	Advantages and Disadvantages
Pipeline	Separate subtasks	Propagation error and reductant entities prediction
Parameter sharing	Merge subtasks	Void propagation error but cause computing resources problem
Sequence tagging	Merge subtasks	Reduce resources waste but need high-quality data
Sequence tagging and active learning	Merge subtasks and active learning	High-quality data problem is alleviated

**Table 2:** Comparison between different DAL models

Models	Task	Characteristics	Effectiveness
CNN-CNN-LSTM	NER	DAL	Verified the performance of DAL method on NER
Word2vec and BERT	NER and RE	DAL	Verified the performance of DAL method on RE and NER
CNN-CNN-LSTM	NER	Subsequence-based DAL	Verified the performance of querying subsequences on NER
BERT-CRF(SDSAL)	RE	Subsequence-based DAL	Verified the performance of subsequences and distant supervision on RE

Relation extraction methods are mainly divided into two categories: pipeline methods and joint methods [13, 14]. Table 1 shows the comparison between different RE methods. The pipeline method splits entity recognition and relationship classification into two separate tasks with sequential order [15]. The entities are extracted from the text, then the relationships between the entities are classified. As a result, this approach suffers from redundant entity prediction and error propagation.

The other method is joint extraction approach which treats entity recognition and relationship classification as one simultaneous task [16]. Since the joint method can capture the joint features between entity and relationship, it seemly gives better performance [17]. There have been numerous models proposed for joint entity and relation extraction. Among these methods, sequence tagging approach has received the most attention as only one model is needed to extract both entities and relationships between entities [18]. Sequence tagging method depends heavily on quality of large amount of labeled corpus. However, creating high quality labeled datasets is expensive because it takes a lot of prior knowledge to complete annotating tasks.

The problem has been much discussed by active learning algorithms in recent literature [19]. In order for the model to learn more meaningful information, traditionally these methods use sampling strategy to select sequences that are close to decision boundaries, where uncertainty is high. For example, Agrawal [20] gives a modified least confidence-based sampling strategy, Marcheggiani [21] investigates minimum token margin(MTM) strategy that is a variant of the margin sampling strategy, or Balcan [22] offers the maximum token entropy(MTE) measure to the ambiguity about the label of a token. In addition, Bayesian uncertainty estimation method is conducted in [23]. These techniques ensure to choose crucial samples for training and minimize labeling cost.

As deep learning methods have yielded impressive results in many areas, some of the research has focused on deep active learning(DAL) methods. Table 2 shows the comparison between different DAL models. In [24], CNN-CNN-LSTM is proposed in named entity recognition tasks based on active learning. By using a two-layer CNN as an encoder, a ReLU nonlinear transform and Dropout [25] between the two CNN layers, and a direct LSTM as a decoder for anticipating the label sequences, the model successfully represents a textual representation confirming performance using the publicly accessible dataset OntoNotes$-$5.0. The authors of [26] introduce an explainable active learning that combines methods word2vec models and transformer-based BERT models, and model learns static and dynamic contextual embeddings. The method processes the relation extraction for the coronavirus disease 2019 (COVID-19). The above models are several successful attempts of combining deep learning with AL for sequence tagging tasks. However, the authors only consider labelling the full sentences.Besides, some works have not used active learning, but they have made improvements in the pre-training language models and got good results. In [27], Chinese-RoBERTa-wwm-ext-large is proposed in relation extraction tasks and the pre-training language model showed good performance. In another work [28], they proposed a Multi-bert-wwm model by changing the structure of BERT which also showed good performance.

In [29], the authors allow the AL algorithm to query subsequences within sentences, and then experiment with CNN-CNN-LSTM model for NER task. They also give uncertainty analysis for both full sentence and subsequence querying, and it achieves improvements compared to querying full sentences. This work follows the approach of using subsequences and training on partially labeled sentences, and constructs Chinese-RoBERTa-CRF model for relation extraction of clinical texts. It provides a subsequence tagging of unlabeled texts via distant supervised dictionary matching. This further lowers the overall annotation effort by enabling the spread of these labels to identical subsequences in the dataset. We validate our methods, and also evaluate sampling strategies and deep learning models.

## Methods

This section presents our method and core contributions. It is difficult for announcers without medical research background to understand the professional knowledge of medical data sets, so the annotation cost of medical relationship extraction data sets is relatively expensive. For this problem, the active learning strategy is adopted to alleviate the time-consuming, laborious and costly problem of manually labeling medical relationship extraction dataset. Traditional active learning takes a complete sample as the sampling unit. For the task of medical relationship extraction, this method has certain disadvantages. If you sample at the complete sentence level, it may lead to a waste of labeling resources. A more flexible active learning algorithm is introduced. Radmard [29] use the AL algorithm to query subsequences within sentences, their experimental results on NER task confirmed the validity of subsequences relative to complete sequences. This work follows the approach of using subsequences and training on partially labeled sentences. On the basis of this method, text pre-annotation is carried out with remote supervision, which further reduces the amount of manual annotation. This is achieved by training an active learning-friendly relation extraction model, scoring and querying subsequences within sentences, and matching and tagging subsequences by distant supervision.

**Fig. 1:**
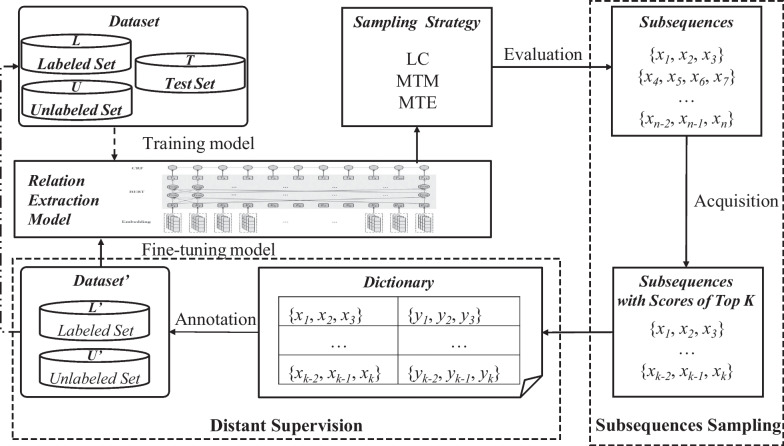
The flow chart bmp of subsequence and distant supervision based active learning

In particular, after initially training a relation extraction model with a small pool of labeled examples, subsequences were queried with scores of Top K based on the uncertainty sampling strategy. Then, the texts and labels of the subsequences are put into the dictionary when the manual annotation is completed. Finally, using the distant supervision mode, the remaining unlabeled subsequences are annotated remotely using dictionary text matching. The training set is updated and the model is fine-tuned at the same time. The above steps are repeated until the number of iterations reaches a predefined value. The flow chart shows in Fig. 1. The method proposed used an active learning strategy based on subsequence sampling to alleviate the labeling pressure on the medical text relational extraction dataset and remote supervision is used to further reduce the labeling amount. To prove the effectiveness of the method, relation extraction model is used to conduct experiments on the CMeIE dataset to compare the result of three different strategies.

### Relation extraction model

**Fig. 2:**
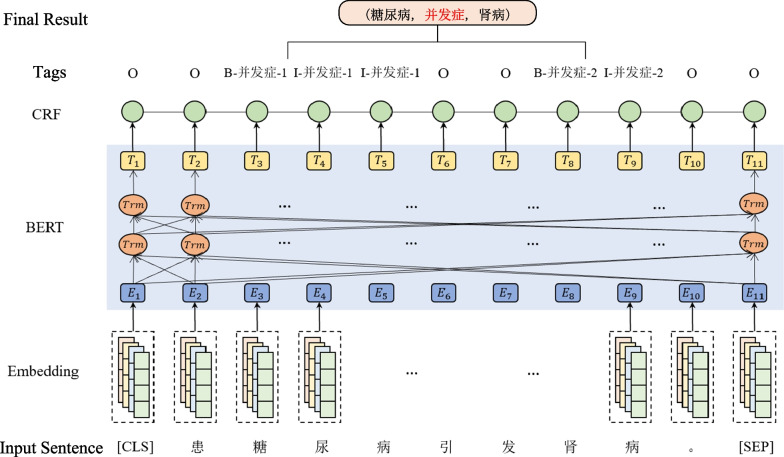
Overall framework of RE model based on RoBERTa-CRF

In comparison to other models, the Chinese-RoBERTa-wwm-ext-large model is Chinese BERT with Whole Word masking [30] and can learn rich semantic contextual features and especially good for Chinese language processing, whereas the Conditional Random Field(CRF) [31] can capture context annotation information [32]. Therefore, it is advisable for us to apply a Chinese-RoBERTa-CRF model to Chinese medical texts for relation extraction. Figure 2 summarizes the model architecture. It blends Embedding layer, BERT layer, and CRF layer. The Embedding layer firstly takes the original text sequence and transforms it into a sequence of character vectors. Then, the Chinese-RoBERTa per-trained language model is used to learn representation of characters. Finally, to output the label for each character in the sequence, a CRF layer is utilized as the decoder.

The tagging scheme [33] is defined by the “BIO” (Begin, Inside, Outside) signs. Since the connection role should be included in the label in the relation extraction task, the digits ”1” and ”2” are used to indicate whether the character belongs to the head or tail entity. The tag of the input sentence “患糖尿病引发肾病” [Diabetes causes nephropathy.] is “O B-并发症-1 I-并发症-1 I-并发症-1 O O B-并发症-2 I-并发症-2 O [O B-complication-1 I-complication-1 I-complication-1 O O B-complication-2 I-complication-2 O]”. Finally, an extracted result is represented by a triplet “(糖尿病,并发症,肾病)[(diabetes, complication, nephropathy)]”, in other words the relation “并发症[complication]” is recognized. If the input sentence is ”Heart failure causes pneumonia.”, the tag will be “B-complication-1 I-complication-1 O B-complication-2 O” and the extracted result will be (Heart failure, complication, pneumonia).

### Subsequence acquisition

Uncertainty Sampling is motivated by an assumption that if the model is uncertain about the instance label then the instance might be from an unexplored region of the feature space or it lies on the decision boundary of a classifier. Therefore, such instance might be crucial for manual annotation. The common uncertainty-based sampling methods [34], such as Least Confidence (LC) [35],MTM, and the MTE, can be applied to quantify the uncertainty for variable length subsequences. The LC score is defined as:

1 $$\begin{aligned} S^{LC}(x)=1-p(y^*|x) \end{aligned}$$

where $y^*$ is the label of max probability. The MTM score is then defined as

2 $$\begin{aligned} S^{MTM}(x)=-\sum _{i=1}^{N} p(y_i|x)\cdot \log p(y_i|x), \end{aligned}$$

where *N* denotes the type numbers of the labels and $y_i$ is the label of the *i*-th type, and for MTE as

3 $$\begin{aligned} S^{MTE}(x)=p(y^{*}|x)-p(y^{'}|x), \end{aligned}$$

where $y^{'}$ is the label of the second max probability. Then, both sequences and subsequences uncertainty scores are generalized by simply defining:

4 $$\begin{aligned} Uc(X)=\frac{S(x_1)+S(x_2)+...+S(x_l)}{l} ,\end{aligned}$$

where *l* is the length of *X*.

The greedy algorithm is used to select subsequences [29]. Further, there is no overlap with previously selected subsequences, i.e., there is no intersection between text positions. Additionally, the length of the selected subsequence can be adjusted in the actual situation. For example, it is feasible to set a subsequence length window and limit its size to $[l_{min}^s,l_{max}^s]$ and only query subsequences within this range.

**Fig. 3:**
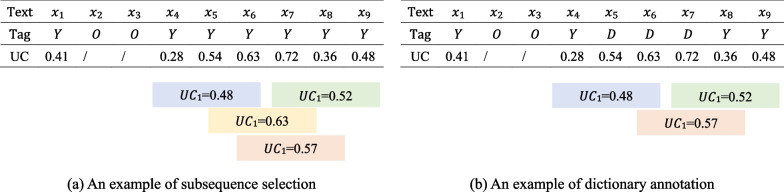
An example of subsequence acquisition using $l_{max}^s=l_{min}^s=3$

An example of how to select a subsequence is shown in Fig. 3a. The lengths are restricted to $l_{max}^s=l_{min}^s=3$ to illustrate the approach of selecting subsequences in a straightforward and intuitive manner. A sample X with length 9 is given, which denotes as X= $\{ x_1,x_2...,x_{9}\}$. Label O represents that the character has been labeled by an expert and Y represents that the character has not been labeled and needs to be labeled based on the sampling mechanism. UC means uncertainty, its size will determine whether labeling is required. Different colors are used to distinguish different subsequences. For example, the blue part represents the mean uncertainty of the subsequence {$x_4,x_5,x_6\}$ and the green part represents the mean uncertainty of the subsequence $\{x_7,x_8,x_9\}$. In this scenario, the subsequences $x_2$ and $x_3$ have been labeled, and they will not be considered in the selection process. Then, the subsequences with lengths of three and without annotation are traversed, and the uncertainty score of each subsequence is determined based on the sampling mechanism. The subsequence with the highest uncertainty score is chosen for manual annotation. Repeat the process until the total of subsequences is selected.

In Fig. 3a, the subsequence {$x_5$, $x_6$, $x_7$} has the maximum uncertainty score, which is 0.63. As a result, this subsequence is chosen for manual annotation. However, once the annotation of {$x_5$, $x_6$, $x_7$} is finished, the text will have no one unlabeled subsequence of length 3. There will be just one subsequence {$x_1$, $x_4$} of length 2, and two subsequences {$x_1$}, {$x_4$} of length 1. Since there is a total of three subsequences to choose from in this iteration, two more subsequences must be chosen. The length constraint can now be eased to allow for the selection of shorter subsequences. The subsequences {$x_8$, $x_9$}, and{$x_1$} will be selected one by one in this example.

### Dictionary annotation based on distant supervision

To further reduce the total annotation effort, this paper presents a dictionary annotation method based on distant supervision strategy. It propagates manually annotated subsequence labels to unlabeled subsequences. Since the sampling unit is the subsequence, the annotator does not acquire its contextual information in the remainder of the sentence. When textually consistent subsequences are given, the annotator will make the same labels.

In an iterative process of active learning, the method maintains a dictionary that maps the text of previously annotated subsequences to the labels to which they belong, and then assigns the same temporary labels to all matching subsequences in the unlabeled set based on lexical pattern matching. When selecting subsequences, the uncertainty of subsequences is already in the dictionary. It is no longer calculated as the labeled set, i.e., subsequences that are identical to the text in the dictionary is no longer considered. However, if $Sub_A$ appears in the dictionary as part of a different subsequence $Sub_B$, the uncertainty of $Sub_B$ is calculated. And once $Sub_B$ is selected for manual annotation, the temporary dictionary label for the $Sub_A \cap Sub_B$ part of the text is overwritten and the text of $Sub_B$ and its label are added to the dictionary. Its tags will be added to the lexicon, which will be updated after each iteration.

Figure 3b illustrates the process of dictionary annotation through an example. In this example, the subsequence {$x_5, x_6, x_7$}, which has been handed over to manual annotation during the previous iterations, is added to the dictionary. In this iteration, {$x_5, x_6, x_7$} reappears in the text, and {$x_5, x_6, x_7$} is given a temporary label based on lexical pattern matching using distant supervision. The label ’D’ in the example means that the character’s label is marked by the dictionary.

Since {$x_5, x_6, x_7$} already existed in the dictionary, this subsequence is not considered in this iteration. Uncertainty is calculated for the remaining subsequences that have not been manually annotated. In this example, the uncertainty score of the subsequence {$x_6, x_7, x_8$} is the highest. Although {$x_6, x_7$} have been remotely labeled by the dictionary, the tags labeled by the dictionary are only temporary, and {$x_6, x_7, x_8$} can still be selected for expert labeling. The new tags will be overwritten by the manually annotated tags. If there is no longer a subsequence of length 3 in the text, subsequence of length equal to 2 is selected. In this iteration, subsequences {$x_4, x_5$} and {$x_9$} will be selected in turn. Meanwhile, the lexicon will be updated and the texts and corresponding labels of the subsequences {$x_6, x_7, x_8$}, {$x_4, x_5$} and {$x_9$} will be added to the dictionary, and the remaining unlabeled subsequences will be remotely labeled based on the dictionary.

### Active learning algorithm

The final pseudo-code for the SDSAL method for relational extraction is shown in Algorithm 1.

Given an unlabeled dataset *U* and a labeled dataset *L*, all subsequences that satisfy length restriction and do not exist in the dictionary are identified from the unlabeled dataset and scored based on the uncertainty method, and the length limit can be relaxed if the number of subsequences does not reach a predefined value. Next, the top K scored subsequences are selected for manual annotation. When the manual annotation is completed, the text of the subsequences and their corresponding labels are saved in the dictionary *Dict*. Then, the subsequences are merged into the annotated set and deleted from the unlabeled set. Next, the unlabeled set is remotely pre-labeled with *Dict* based on text matching, and the new labeled set is used to train the Chinese-RoBERTa-CRF model and evaluate its *F*1 value on the test set. Eventually, the above process is iterated until the number of iterations reaches a predefined value.
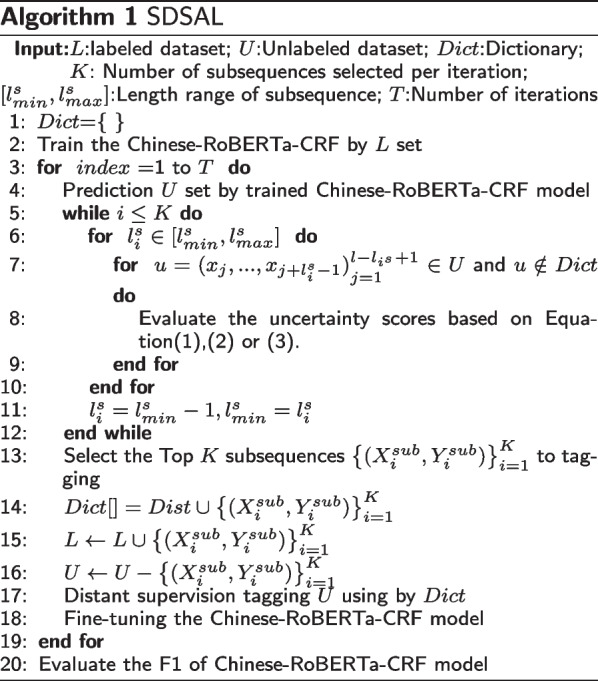


## Experiments

### Experimental setting

The largest schema-based Chinese medical information extraction dataset Medical Entity Extraction dataset (CMeIE)^1^ is used for experiments [36]. The dataset is a relatively standard Chinese medical information extraction dataset that is open source in China. In addition, many papers used this dataset to verify the effectiveness of their relationship extraction methods. This dataset is created through multiple iterations of manual annotation and includes 28,008 sentences, 85,282 triplets, 11 entities, and 44 relations taken from clinical notes and medical literature. It is appropriate for NER and RE task in the Chinese medical domain [28, 37].

Considering the number of relationships and their distribution in the dataset, the sentence of ”disease_disease” is extracted as the final dataset for the experiment. With 2,335 sentences in the training set and 604 sentences in the testing set, the training set is divided into an initial labeled set and an unlabeled set in the ratio of 2:8. Table 3 shows the detail information of the dataset.

**Table 3:** Statistics of different relation types in our dataset

Relation type	Training set	Labeled set	Unlabeled set	Testing set
Complications	2057	447	1610	541
Pathological subtypes	1848	351	1497	497
Associated (leading to)	1496	284	1212	315
Differential diagnosis	1331	245	1086	290
Related (transformation)	718	173	545	165
Related (symptoms)	429	76	353	74

### Evaluation metrics

Precision, Recall, and F1-value are used to evaluate the effectiveness of our suggested model. The formulas are shown below.

5 $$\begin{aligned} Precision= & {} \frac{TP}{TP+FP} \end{aligned}$$

6 $$\begin{aligned} Recall= & {} \frac{TP}{TP+FN} \end{aligned}$$

7 $$\begin{aligned} F1= & {} \frac{2 \times Precision\times Recall}{Precision+Recall} \end{aligned}$$

Precision measures a model’s capacity to present only accurate entities, recall measures a model’s capacity to identify every entity in a dataset, and F1-value is the harmonic mean of precision and recall.

### Hyperparameters

The hyperparameters used in this model are shown in Table 4. The maximum sequence length is fixed at 128; the batch size for the training phase of the model is set to 36; the optimizer is Adam, and the learning rate is set to $5*10^{(-6)}$. The graphics card used in the experiment is GTX 3090 and the training time for each strategy takes about 4 h. The code used in the experiment is based on tensorflow and keras.

**Table 4:** Parameter configuration of our model

Hyperparameter	Value
Size of sequence	128
Batch size of training set	36
Optimizer	Adam
Learning rate	$5*10^{-6}$

In the active learning process, the number of iterations is set to seven, and 450 subsequences are chosen in each iteration. The size of the subsequences was restricted between 40 and 45 because the mean, median, and plurality were 44.12, 38, and 14 correspondingly.

## Results and discussion

### Comparative for different model

For assessing the effectiveness of Chinese-RoBERTa-CRF on tasks requiring the extraction of medical relation, this section conducts experimental comparisons with various common models containing CNN-CNN-LSTM, BiLSTM-LSTM, BERT-LSTM and BERT-CRF. By contrasting BERT-LSTM with BERT-CRF, the decoder performance of LSTM and CRF is examined.

After all five models had been trained using the whole training set, the effects are verified on the testing set. Table 5 presents the comparison results.

**Table 5:** Comparative results of different models for extraction relation

Model	Precision	Recall	F1 value
CNN-CNN-LSTM	39.62	26.07	31.25
BiLSTM-LSTM	44.55	34.56	38.65
BERT-LSTM	49.66	53.4	51.28
BERT-CRF	52.58	52.05	52.13
Chinese-RoBERTa-CRF	**58.02**	**53.84**	**55.45**

Table 5 demonstrates that Chinese-RoBERTa-CRF obtained the highest precision, recall and F1 values with 58.02% 53.84% and 55.45% respectively and the scores are bold in the table. BERT-CRF had the second highest precision and F1 values with 52.58% and 52.13% respectively. The comparison between Chinese-RoBERTa-CRF with BERT-CRF shows the good encoder performance of Chinese-RoBERTa. BERT-LSTM was in the third position with precision and F1 values of 49.66% and 51.28% respectively, while CNN-CNN-LSTM scored the lowest with 39.62%, 26.07% and 31.25%.

On the encoder side, it can be observed that the F1 values of BERT-LSTM are 12.63% and 20.03% higher than those of BiLSTM-LSTM and CNN-CNN-LSTM, respectively. BERT understands the semantic information of the text more effectively than BiLSTM because it obtains the positional representation of the text through positional encoding and captures the correlation between each word based on a self-attentive mechanism. Similarly, BiLSTM learns contextual representations from a deeper perspective by forward and backward encoding of sequences through bi-directional LSTM, and is therefore better at extracting long distance dependencies compared to CNN-CNN model.

On the decoder side, it can be found that the F1 value of BERT-CRF is 0.85% higher than that of BERT-LSTM. The reason for this may be that CRF is based on the idea of dynamic programming and uses the Viterbi algorithm for decoding [38]. Compared to LSTM, CRF takes into account the constraint relationships between labels through the transfer probability of labels, and is therefore more suitable for the task of medical relationship extraction based on sequence tagging. And the comparison result between BERT and Chinese-RoBERTa shows that the latter is more appropriate. In summary, Chinese-RoBERTa-CRF model is suitable relation extraction model in Chinese medical texts.

### Comparative for existing methods

To assess the effectiveness of the SDSAL proposed, this section compares the SDSAL with the following two models.

1. Subsequence based Active Learning (SAL): It uses subsequences as sampling units for active learning, although it does not use dictionaries for remote annotation.
2. Full sequence based Active Learning (FAL): It uses a complete sequence as the sampling unit for active learning and does not use a dictionary for remote annotation.

**Fig. 4:**
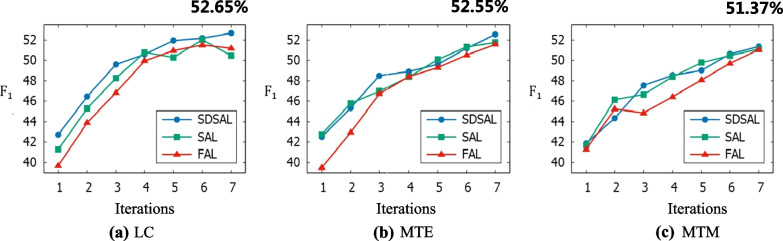
Comparisons between different methods based on three sampling strategies

Among them, comparing SDSAL with SAL can verify the effectiveness of distant supervision, while by comparing SAL with FAL, the superiority of subsequence sampling can be demonstrated. Three sets of experiments were conducted based on the three sampling strategies introduced in "Subsequence acquisition" section, namely LC, MTE and MTM, and the comparison results are shown in Fig. 4.

Figure 4 shows the performance of the BERT-CRF model under all sampling methods. It indicates that the increase in training data enables the relation extraction model BERT-CRF to fully learn the feature representation of the texts, thus improving the performance of identifying relationships between entities. In Fig. 4a shows the performance of the three models based on the LC sampling strategy. The F1 value of SDSAL increases as the number of iteration rounds increases. During the first iteration, the F1 values of all three methods increased rapidly. And in the later iteration process, SDSAL and FAL gradually levelled off, while the F1 values of SAL stopped increasing in the fifth and seventh rounds, and decreased significantly instead. It probably because the features of the selected subsequences in these two rounds are too complex to cause the model to overfit. Overall, SDSAL is the most effective based on the LC sampling strategy. The performance gap between the three methods is more obvious, verifying the effectiveness of our method.

Therefore, it can be observed that the F1 values of all three methods increase with the number of iterations based on the MTE sampling strategy in Fig. 4b. The small difference between the F1 values of SDSAL and SAL in most iteration rounds indicates that the MTE sampling strategy tends to choose subsequences that overlap with the dictionary, thus covering the labels remotely labeled by the lexicon. Although the difference is not significant, it can be seen that the final F1 value of SDSAL is still higher than that of SAL, so the importance of remote supervision cannot be ignored. Furthermore, it can be seen that the gap between FAL and SDSAL and SAL is larger in the early stages and narrows significantly during the later iterations, indicating that the samples selected by FAL in the later stages contain information-rich subsequences and thus the performance is improved.

From Fig. 4c, the F1 values of all the methods keep increasing as the number of iterations increases, except for FAL where the F1 value decreases at the third round. The gap between SDSAL and SAL remains small, similar to the effect of the MTE sampling-based strategy. It indicates that there is an overlap between the subsequences selected by MTM and the annotation of the dictionary, resulting in the remote annotation of the dictionary labels being overwritten. However, the performance gap between FAL and SDSAL and SAL is more significant, indicating that there is heterogeneity in the uncertainty of subsequences in the samples selected by FAL. i.e., the relation extraction model BERT-CRF can make correct predictions for the labels of some subsequences in the sample, while for others it is uncertain. Furthermore, SDSAL and SAL effectively improve the performance by selecting subsequences with higher annotation values in the sample, which validates the superiority of selecting subsequences.

In order to quantitatively analyze the different effects of SAL, FAL and SDSAL, this section takes the highest F1 value in the iteration process of each method based on different sampling strategies as its final result, and records the iteration rounds when the highest F1 value is reached.

**Fig. 5:**
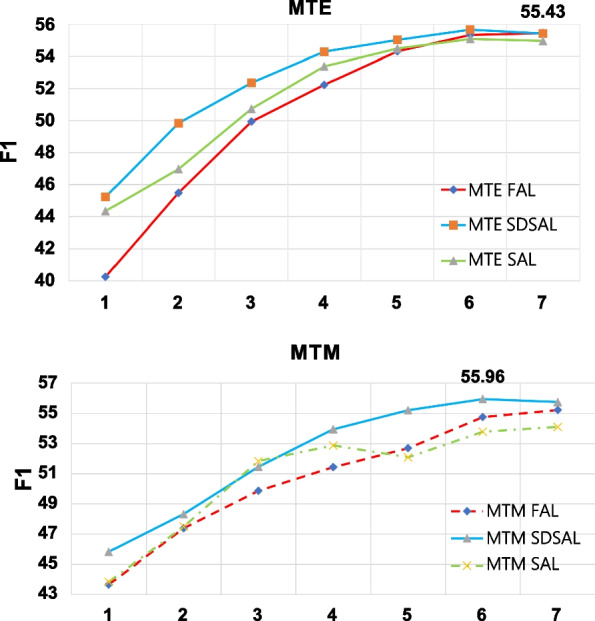
Chinese-RoBERTa-CRF methond on MTM and MTE strategy

From the comparison results it can be seen that whether based on LC, MTE or MTM strategies, SDSAL achieved the highest F1 values, 52.65%, 52.55% and 51.37% respectively. It is evident that SDSAL outperforms SAL in terms of results. It demonstrates that the distant supervision further enhances the model by performing dictionary matching on the remaining subsequences of the unlabeled set, and also confirms the efficacy of distal supervision. Besides, the advantage of SAL over FAL suggests that identifying information-rich subsequences in the sequence can effectively boost the relation extraction model’s performance. As a result, the effectiveness of subsequence tagging is confirmed.

Further, except that FAL and SAL get the maximum F1 value in the sixth iteration based on LC strategy, the best outcomes are obtained in the last round. It illustrates that the method got better results as the training set increased.

It shows that the F1 value of BERT-CRF is 52.13% when it is trained based on the full training set. Almost all approaches produce outcomes that are comparable to those utilizing the entire training set.Moreover, SDSAL even generates results that are superior to those of the entire training set. This demonstrates that active learning techniques based on subsequence and distant supervision can find information-rich subsequences, which can further increase annotation efficiency.

### Effectiveness on Chinese-RoBERTa-CRF

In order to analyze the effects of Chinese-RoBERTa-CRF model on SAL, FAL and SDSAL, this section makes experiments on these sampling strategies and the results are shown in Fig. 5.

Figure 5 shows the results of relation extraction when the Chinese-RoBERTa-CRF model uses MTM and MTE sampling strategies. It shows that with the increase in the number of iterations, the effect of SDSAL is obviously better than the other two strategies and reaches the peak of 55.96% and 55.43%,which is 4.59% and 2.88% higher than BERT-CRF. The result also shows the better performance of Chinese-RoBERTa pre-trained model in processing Chinese texts. Besides, both experiments of BERT-CRF and Chinese-RoBERTa-CRF on SDSAL sampling strategy have achieved good results. The proposed method has certain robustness.

## Conclusion

We have seen a combining subsequence querying and distant supervision method for relation extraction tasks. The proposed SDSAL technique increases the relational extraction model’s performance as well as the annotation efficiency by refining the sampling unit and selecting highly informative subsequences. Meanwhile, the unlabeled set is remotely pre-annotated using text matching based on the concept of distant supervision. It further minimizes the annotation cost. The experimental results show that our method achieves the highest F1 value compared to existing benchmark methods on the CMeIE dataset.

In the real world, the process of manual annotation may lead to inaccurate annotations due to insufficient contextual information on the subsequences. Therefore, the issue of how to handle these loud labels needs to be researched further in the future.

## Data Availability

The data and code that support the findings of this study are available from https://github.com/yehqi/RelationExtraction.

## Footnotes

1 https://tianchi.aliyun.com/dataset/dataDetail?dataId=113223.
